# Factor structure of the Parenting Stress Index-Short Form used in the Japan Environment and Children’s Study

**DOI:** 10.1038/s41598-022-23849-8

**Published:** 2022-11-09

**Authors:** Takehiro Hatakeyama, Kenta Matsumura, Akiko Tsuchida, Hidekuni Inadera, The Japan Environment and Children’s Study (JECS) Group, Michihiro Kamijima, Shin Yamazaki, Yukihiro Ohya, Reiko Kishi, Nobuo Yaegashi, Koichi Hashimoto, Chisato Mori, Shuichi Ito, Zentaro Yamagata, Takeo Nakayama, Tomotaka Sobue, Masayuki Shima, Hiroshige Nakamura, Narufumi Suganuma, Koichi Kusuhara, Takahiko Katoh

**Affiliations:** 1 Toyama Regional Center for JECS, University of Toyama, 2630 Sugitani, Toyama, 930-0194 Japan; 2 Department of Public Health, Faculty of Medicine, University of Toyama, Toyama, Japan; 3 Nagoya City University, Nagoya, Aichi Japan; 4 National Institute for Environmental Studies, Tsukuba, Japan; 5 National Center for Child Health and Development, Tokyo, Japan; 6 Hokkaido University, Sapporo, Japan; 7 Tohoku University, Sendai, Japan; 8 Fukushima Medical University, Fukushima, Japan; 9 Chiba University, Chiba, Japan; 10 Yokohama City University, Yokohama, Japan; 11 University of Yamanashi, Chuo, Japan; 12 Kyoto University, Kyoto, Japan; 13 Osaka University, Suita, Japan; 14 Hyogo Medical University, Nishinomiya, Japan; 15 Tottori University, Yonago, Japan; 16 Kochi University, Nankoku, Japan; 17 University of Occupational and Environmental Health, Kitakyushu, Japan; 18 Kumamoto University, Kumamoto, Japan

**Keywords:** Psychology, Health care, Health occupations

## Abstract

The Parenting Stress Index-Short Form (PSI-SF) has been widely employed to assess parenting stress in a number of research and clinical trials. To date, no parenting stress studies in Japan have examined the factor structure, validity, and reliability of the PSI-SF. Therefore, this study aimed to evaluate the psychometric properties of this 19-item version as administered in a national cohort study, the Japan Environment and Children’s Study, to two sample groups of mothers, those with a 1.5-year-old child and those with a 2.5-year-old child (n = 79,282 and 75,831, respectively). We performed exploratory factor analysis to re-examine the appropriate factor structure, confirmatory factor analysis to evaluate goodness of fit, and calculated Cronbach’s α and Pearson’s r coefficients to evaluate internal consistency and reproducibility over time, respectively. The results highlighted that a three-factor structure fit the instrument better than a two-factor structure, yielding better scores for the model fit indices and the α and r coefficients. In addition, the third factor identified in this study was strongly associated with having a relationship with and help from the husband. The findings suggest the importance of using a parenting stress scale with various factors to evaluate mothers’ parenting stress.

## Introduction

Parenting stress is distress or discomfort caused by childrearing demands that parents experience or by the parents’ inability to meet these demands and manage parenting stressors through regular family-coping strategies^1^. Parenting stress can be negatively associated with parent–child well-being and general health, such as adversely affecting the quality of the parent–child relationship, the child’s behavior and development, and the parent’s mental health^2–4^.

A systematic review investigating the stress level experienced in parenting revealed that various studies employed a variety of measures to assess parenting stress^5^. For example, eight studies used the Parental Stress Scale, six used the Family Impact Questionnaire, six used the Caregiver Strain Questionnaire, and 14 used other measures. Remarkably though, 102 studies employed the Parenting Stress Index (PSI) or its abbreviated version, the Parenting Stress Index-Short Form (PSI-SF), developed by Abidin^5–9^.The PSI consists of 101 items for identifying parent–child relationships under stress and indicating their stressors^6–8^, while the PSI-SF is condensed to 36 items to make the instrument more convenient to use in research and clinical trials^9^. It appears then that the PSI and PSI-SF are the most widely used measures in parenting stress studies.

The PSI-SF relies on a three-factor structure, with the subscales “Parental Distress (PD-SF),” “Parent–Child Dysfunctional Interaction (PCDI-SF),” and “Difficult Child (DC-SF)^9^.” The PD-SF subscale evaluates the parent’s experienced distress in being a parent, the PCDI-SF subscale evaluates the parent’s perception of the extent to which (or whether) their satisfaction or expectations in parenting are met, and the DC-SF subscale evaluates the degree of the parent’s distress resulting from the child’s difficult behaviors^9^. Other versions of the PSI-SF that support Abidin’s three-factor structure have been widely used in various populations in many countries and regions, including the followings: parents of children with autism spectrum disorders (ASD), parents from minority communities, and low-income Hispanic mothers in the U.S.A.; mothers of children with behavioral problems and from a general population in Spain; low income mothers in Chile; parents of children with ASD in France; and parents from general populations in India, Thailand, and Hong Kong^9–17^. In addition, the PSI-SF that relies on a two-factor structure to evaluate parenting stress associated with parenthood and childrearing has also been used with parents from a general population in the USA, mothers from at-risk families in Spain, and mothers of children with cochlear implants in Taiwan^3,18,19^.

Most of these studies present plausible results on the reliability and validity of the PSI-SF in various sociocultural contexts. For example, internal consistency with Cronbach’s α of total stress level was found to range from 0.83 to 0.93^3,10–12,14,15,19^ on top of the initial PSI-SF (α = 0.91)^9^. In terms of concurrent validity, Reitman et al. revealed associations of PCDI-SF score with Oppositional scale score on Conners’ Parent Rating Scale–Revised: Long Form (β = 0.30, *p* < 0.01) and of PD-SF score and DC-SF score with Mothers’ scores on the Brief Symptom Inventory (β = 0.32, *p* < 0.005 and 0.46, *p* < 0.001, respectively)^20^. Haskett et al*.* assessed construct validity by comparing a first factor comprising the PD-SF and a second factor that consisted of the PCDI-SF and DC-SF with similar scales to measure parent’s emotional health (Global Severity Index), child behavior (Eyberg Child Behavior Inventory), and parenting behavior (Conflict Tactics Scale)^19^. They found associations of the first factor with Global Severity Index score (*r* = 0.54, *p* < 0.001) and of the second factor with Conflict Tactics Scale score (*r* = 0.23, *p* < 0.01) and Eyberg Child Behavior Inventory score (*r* = 0.61, *p* < 0.001). They also found that the PSI-SF showed high test–retest reliability over a 1-year period; the reliability score of the first factor was *r* = 0.61 (*p* < 0.005), while that of the second score and of total score was each *r* = 0.75 (*p* < 0.001). Several studies have developed and employed different versions of the PSI-SF and assessed the psychometric properties, including the 15-item^21,22^, 21-item^10^, and 24-item^23^ versions. As with the factor structure of the original 36-item version^16^, most of these studies found that the three-factor structure better fit their PSI-SFs than other factor structure models, such as one- or two- factor models. In Japan, Araki et al*.*^24^ developed the 19-item PSI-SF to evaluate postpartum parenting stress based on the 78-item PSI proposed by Narama et al*.*^25^, which had originated from Abidin’s original 101-item PSI. This 19-item PSI-SF is designed to evaluate mothers’ parenting stress level related to childrearing at 1.5 years of age^26^, which is the age at which mothers’ parenting stress and anxiety tends to show an increase on the DC-SF and when a routine mother–child checkup can act as a barometer for prediction and intervention^24,25,27^. The factor structure of the 19-item PSI-SF is represented by the two PD-SF and DC-SF subscales.

Given the reliable applicability of a three-factor-structure PSI-SF with different components and in various populations, we hypothesized that the same would be found for the 19-item PSI-SF in a large Japanese population sample. To this end, this study sought to evaluate the factor structure of the 19-item PSI-SF as an assessment of postpartum parenting stress among mothers of a child at 1.5 years of age or 2.5 years of age from a general population in Japan. The instrument was employed in a nationwide birth cohort study, the Japan Environment and Children’s Study (JECS), which investigates associations between various environmental factors and children’s health, and we analyzed large samples for the two different age groups (n > 75,000, respectively). We first examined the factor structure of this instrument, carrying out both exploratory factor analysis (EFA) and confirmatory factor analysis (CFA) while assessing its reliability and validity. We then calculated Cronbach’s α of the factors derived and compared their Pearson’s correlation coefficients and factor scores between the two parenting periods.

## Methods

### Participants

Participants were mothers who took part in the JECS. Recruitment for the study occurred in 15 regional centers across Japan from January 2011 to March 2014 and involved a face-to-face explanation of the survey for potential participants. Details of the JECS design are described elsewhere^28,29^. All procedures contributing to the JECS comply with the ethical standards of the relevant national and institutional committees on research involving human participants and with the Helsinki Declaration of 1975, as revised in 2008. The JECS protocol was reviewed and approved by the Ministry of the Environment’s Institutional Review Board on Epidemiological Studies and the ethics committees of all participating institutions. All procedures involving human participants in the JECS and the present study were approved by the Ministry of the Environment’s Institutional Review Board on Epidemiological Studies (No. 100910001), the ethics committees of all participating institutions, and the Ethics Committee of the University of Toyama (No. R2022025). Written informed consent was obtained from all participants.

### Data

**Figure 1:**
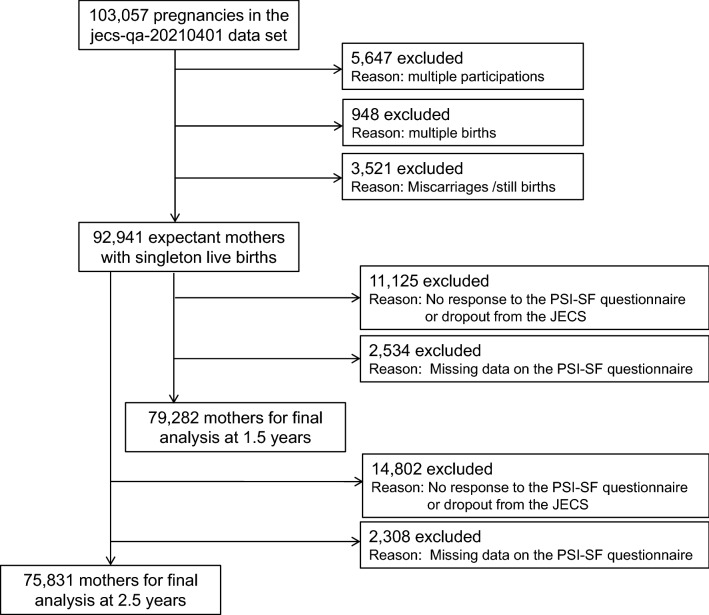
Flow diagram of the study.

We used the dataset *jecs-qa-20210401*, which was released in April 2021 and contains 103,057 registrants. We arrived at the number of unique mothers who participated for the first time by excluding 5647 pregnancies due to multiple registrations, 948 pregnancies due to multiple births, and 3521 pregnancies due to miscarriage or stillbirth, leaving 92,941 unique mothers with singleton live births. In addition, at the 1.5-year and 2.5-year parenting periods, we respectively excluded 11,125 mothers and 14,802 mothers due to no response to the PSI-SF questionnaire or drop out from the JECS and further excluded 2534 mothers and 2308 mothers owing to missing data on the PSI-SF questionnaire. The missing data rates were 3.19% at 1.5 years and 3.04% at 2.5 years, which at less than 5% are considered not to have a significant impact on the results^30^. Those two time periods were selected considering that parenting stress and anxiety increase for mothers with a 1.5-year-old child^24^ and that children become defiant for first time between 2 and 3 years of age, which is associated with parents having higher anxiety and negative feelings about childrearing^31^. Thus, we analyzed data for two groups of mothers with singleton live births, 79,282 mothers with a 1.5-year-old child and 75,831 mothers with a 2.5-year-old child (Fig. 1).

### Measurements

The 19-item PSI-SF, a self-reported questionnaire to measure parent’s postpartum parenting stress level, was administered during the JECS to mothers at the 1.5-year or 2.5-year parenting period. Mothers responded to each question using a five-point Likert-type scale from 1 (strongly disagree) to 5 (strongly agree). This instrument is derived from the 19-item PSI-SF developed by Araki et al.^24^ (Table 1). The developers evaluated the internal reliability of the initial PS-SF, calculating Cronbach’s α as 0.82 for total stress, 0.74 for the PD-SF, and 0.80 for the DC-SF. They also evaluated its criterion validity against the parenting perception and behavior subscales proposed by a previous study^27^ and found *r* values of 0.77 for “Childrearing Stress,” 0.60 for “Negative Childrearing Behavior,” and − 0.52 for “Positive Feelings toward Childrearing”^24^.

**Table 1:** Abbreviated content of questionnaire items for the 19-item Parenting Stress Index-Short Form employed in the Japan Environment and Children’s Study.

Item	Abbreviated content
1	Feeling good at being a parent
2	Resorting to asking people for help or advice
3	Child is active to the extent of overwhelming me
4	Child has difficulty focusing his/her attention
5	Child rarely does things that make me feel good
6	Child cries or fusses rather often
7	Child does not seem to smile much
8	Things that my child does bother me a lot
9	Child is easily upset by small things
10	Child frequently puts demands on me
11	Child is always attached me
12	Feeling incapable of handling things very well
13	Feeling unable to do what I like to do
14	Feeling that something my child does wrong is my fault
15	My husband does not give me as much help as I expected
16	More problems with my husband by having a child
17	Feeling alone without friends
18	Experiencing more illness, aches, and pains over the past 6 months
19	Unable to enjoy things as I used to

### Procedure

#### Exploratory factor analysis

Given that a three-factor structure showed the best model fit to the PSI-SF in several studies, we conducted EFA to re-examine whether a two- or three-factor structure better fit the 19-item PSI-SF data obtained in the JECS at the 1.5-year and 2.5-year parenting periods (n = 79,282 and 75,831, respectively). In the analysis, we employed maximum likelihood extraction with promax rotation. The number of factors were determined using parallel analysis^32^.

#### Confirmatory factor analysis

To evaluate whether the original two-factor structure^18,19^ or a different factor structure identified in the present study better fit the data, we subsequently performed CFA on the same JECS data, using the maximum likelihood estimation procedure. Concurrently, goodness-of-fit of the two- or three-factor structure models were evaluated using the following model fit indices as recommended in previous studies^33–36^: degrees of freedom (df), standardized root mean square residual (SRMR), adjusted goodness-of-fit index (AGFI), root mean square error of approximation (RMSEA), Bentler comparative fit index (CFI), and Bentler-Bonett non-normed fit index (NNFI).

#### Validity and reliability test

We then evaluated the validity of the 19-item PSI-SF of the JECS in terms of its reproducibility, comparing Pearson’s correlation coefficients and factor scores of the derived factors between the two parenting periods, and the reliability of the instrument in terms of its internal consistency, calculating Cronbach’s α coefficients at the two parenting periods. All analyses were performed using SAS software version 9.4 (SAS Institute Inc.).

#### Additional analysis

Finally, we performed an additional goodness-of-fit test on the factor structure model identified by the EFA with the aforementioned model fit indices, using Mplus version 8.4 (Muthén & Muthén). This procedure may yield different estimates to what SAS delivers because the 19-item PSI-SF data relies on a Likert-type scale and Mplus treats the data as categorical variables, whereas SAS treats the data as continuous variables.

## Results

### Descriptive statistics

Participants’ background characteristics were analyzed, given that family income and education can relate to parenting stress and that addiction during the parenting period can cause dysfunctional parent–child interactions^4,20^. Mothers’ mean age was 30.99 (SD, 5.06), 36.16% had less than 12 years of education, 40.10% had an annual income of less than four million yen (approximately 29,000 USD), 3.54% had a regular smoking habit, 2.65% had a regular alcohol drinking habit, and 42.54% were primiparous.

### Exploratory factor analysis

Parallel analysis initially retained a maximum of four factors. However, the factor loading of a variable exceeded one at both parenting points, thus a four-factor structure was an improper solution for the19-item PSI-SF used in the JECS. Alternatively, we found a three-factor structure at both the 1.5-year and 2.5-year parenting periods as follows. Factor 3 was explained by item 15 (parenting stress associated with having a relationship with the husband) and item 16 (parenting stress associated with having help from the husband) with higher factor loadings at each parenting period: 0.83 and 0.95 for item 15 and 0.76 and 0.63 for item 16, respectively (Table 2). Inter-factor correlation coefficients at the respective parenting periods were 0.52 and 0.53 (Factor 1–2), 0.52 and 0.49 (Factor 1–3), and 0.33 and 0.31 (Factor 2–3). The Kaiser–Meyer–Olkin statistic of sampling adequacy was above the minimum criteria of 0.5 for both periods (0.897 at 1.5 years and 0.902 at 2.5 years), and Bartlett’s test of sphericity was statistically significant (*p* < 0.0001), which indicated the correlations among items were not constant at the two parenting periods. Therefore, those items were appropriate for factor analysis.

**Table 2:** Comparison of factor loadings for the three identified factors and mean values with standard deviation (SD) at 1.5 and 2.5 years postpartum.

Items	1.5 years					2.5 years				
	Factor 1	Factor 2	Factor 3	Mean	SD	Factor 1	Factor 2	Factor 3	Mean	SD
1	**0.55**	0.07	− 0.08	1.61	0.67	**0.54**	0.09	− 0.05	1.69	0.69
2	**0.63**	− 0.10	− 0.01	1.80	0.82	**0.62**	− 0.09	0.04	1.83	0.84
7	**0.25**	0.18	− 0.05	1.18	0.45	**0.24**	0.19	− 0.04	1.17	0.42
12	**0.52**	0.23	− 0.01	2.34	1.00	**0.55**	0.23	− 0.03	2.38	1.02
13	**0.43**	0.17	0.12	2.53	0.99	**0.47**	0.14	0.11	2.46	0.97
14	**0.39**	0.15	0.06	2.10	0.89	**0.42**	0.15	0.03	2.14	0.89
17	**0.71**	− 0.07	0.03	1.72	0.87	**0.72**	− 0.07	0.02	1.74	0.88
18	**0.54**	0.01	0.03	1.73	0.98	**0.52**	0.01	0.00	1.81	1.00
19	**0.77**	− 0.02	0.05	1.70	0.86	**0.79**	− 0.03	0.02	1.76	0.90
3	− 0.05	**0.45**	0.03	3.32	1.10	− 0.05	**0.47**	0.03	3.35	1.11
4	0.00	**0.55**	0.00	2.34	0.89	− 0.01	**0.58**	0.01	2.30	0.94
5	0.29	**0.32**	− 0.05	1.40	0.59	0.27	**0.37**	− 0.02	1.45	0.62
6	0.06	**0.65**	− 0.02	1.80	0.88	0.06	**0.63**	− 0.01	1.85	0.90
8	0.13	**0.48**	− 0.01	2.06	1.05	0.13	**0.53**	− 0.03	2.16	1.10
9	− 0.03	**0.68**	0.01	2.25	1.04	− 0.02	**0.69**	0.00	2.28	1.05
10	− 0.02	**0.76**	0.00	2.07	0.99	− 0.03	**0.79**	0.01	2.15	1.03
11	0.04	**0.38**	0.08	2.60	1.07	0.08	**0.34**	0.10	2.43	1.05
15	− 0.04	0.01	**0.83**	2.23	1.14	− 0.06	0.01	**0.95**	2.24	1.13
16	0.09	0.01	**0.76**	2.07	1.09	0.19	0.02	**0.63**	2.08	1.08

Bold type indicates factor loadings contributing to the attributed factors.

### Confirmatory factor analysis

**Figure 2:**
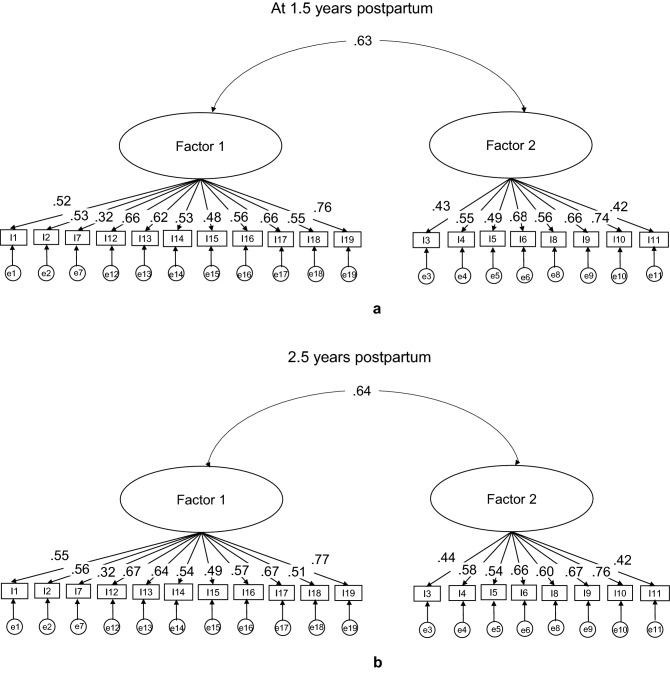

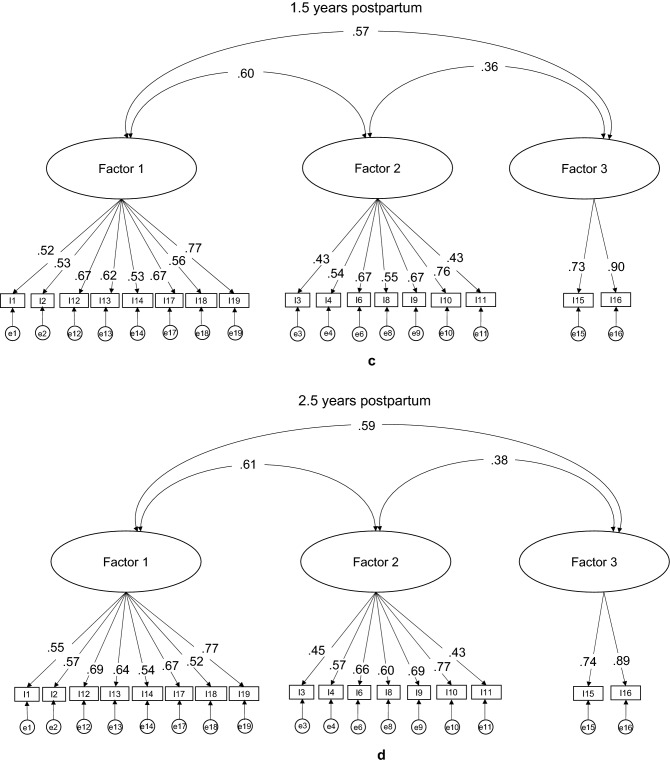
Path diagrams of the two-factor structure (**a**,**b**) and three-factor structure (**c**,**d**) of the 19-item PSI-SF at two parenting periods.

Figure 2 shows path diagrams of the two-factor structure for the original 19-item PSI-SF and the three-factor structure derived by the EFA at the two different parenting periods in this study. Items 5 and 7 were excluded after the EFA because the former almost cross-loaded on Factors 1 and 2, while the latter did not meet the minimum factor loading cut-off point (> 0.32)^37^. Figure 2 also shows that the covariance between Factors 1 and 2 was the same in both the two- and three-factor structures at the two parenting periods (0.63 and 0.64, respectively), while the covariance between Factors 1 and 3 in the three-factor structure showed moderate correlation at the two parenting periods (0.57 and 0.58, respectively). Likewise, estimates for each observed variable indicated similar results in both factor structures at the two parenting periods, while Factor 3 in the three-factor structure was explained at 1.5 and 2.5 years by item 15 (0.73 and 0.74) and item 16 (both 0.90).

Table 3 shows the scores of the model fit indices for the two- and three-factor structures at both parenting periods. We evaluated which factor structure fit the data better in accordance with the following criteria that represent a good model: SRMR close to 0.05, AGFI ≥ 0.90, RMSEA ≤ 0.06, and CFI and NNFI ≥ 0.90^20,33,35,38,39^. Overall, all indices for the three-factor structure had better scores than for the two-factor structure at both parenting periods. Most met the set criteria, with a slightly higher RMSEA value than the criterion and acceptably high NNFI scores of 0.895 and 0.893 at the respective parenting points. Hence, the three-factor structure fit the data better than the two-factor structure.

**Table 3:** Comparative matrix of model fit indices for the two- and three-factor structures at the two postpartum parenting periods.

	1.5 years^a^		2.5 years^b^	
	Two-factor	Three-factor	Two-factor	Three-factor
df(*p*)	151	171	151	149
SRMR	0.060	0.044	0.060	0.045
AGFI	0.878	0.930	0.872	0.924
RMSEA (90% CI)	0.083 (0.082–0.083)	0.063 (0.062–0.064)	0.085 (0.085–0.086)	0.066 (0.065–0.067)
CFI	0.817	0.911	0.882	0.909
NNFI	0.793	0.895	0.797	0.893

*χ*^2^ chi square, *df* degrees of freedom, *SRMR* standardized root mean square residual, *GFI* goodness-of-fit Index, *AGFI* adjusted goodness-of-fit index, *RMSEA (90%CI)* root mean square error of approximation with 90% confidence interval, *CFI* Bentler comparative fit index, *NNFI* Bentler-Bonett non-normed fit index.

a = 79,282 mothers, b = 75,831 mothers.

### Validity and reliability test

Table 4 shows a comparison of factor scores for the three factors between the two parenting periods and of the mean values of all factors that did not show significant differences between the two parenting periods (*p* < 0.0001). Likewise, Table 4 shows the Pearson’s correlation coefficients between the two parenting periods. There is relatively high temporal invariability of Factors 1, 2, and 3 (*r* = 0.73, 0.63, and 0.67, respectively), which are statistically significant (*p* < 0.0001). Hence, reproducibility of the three-factor structure for the 19-item PSI-SF in the JECS was high. In terms of internal consistency, Table 4 shows the standardized Cronbach’s α coefficients at the two parenting periods. All three factors alongside total stress had high scores, ranging from 0.79 to 0.87 at 1.5 years with almost no change a year later at 2.5 years. Hence, reliability of the three-factor structure was high.

**Table 4:** Factor scores, Pearson’s correlation coefficients, and Cronbach's α coefficients at the two postpartum parenting periods.

	Factor score mean (SD)		Pearson's r	Cronbach's α	
	1.5 years	2.5 years	1.5–2.5 years	1.5 years	2.5 years
Factor 1	15.540 (4.757)	15.821 (4.885)	0.730****	0.824	0.831
Factor 2	16.448 (4.572)	16.530 (4.756)	0.623****	0.776	0.789
Factor 3	4.301 (2.027)	4.324 (2.010)	0.671****	0.793	0.799
Total stress	36.226 (9.125)	36.648 (9.489)	0.733****	0.864	0.873

*SD* standard deviation.

****p < 0.0001.

### Additional analysis

AN additional goodness-of-fit test of the three-factor structure revealed fairly similar findings for the two parenting periods: degree of freedom = 116, SRMR = 0.04, RMSEA (90% CI) = 0.071 (0.070–0.072), CFI = 0.947, and NNFI = 0.938 at 1.5 years, and degree of freedom = 116, SRMR = 0.04, RMSEA (90% CI) = 0.075 (0.074–0.075), CFI = 0.945, and NNFI = 0.936 at 2.5 years.

## Discussion

The purpose of this study was to examine whether a three-factor structure fit the Japanese version of the 19-item PSI-SF used in two large sample groups in the JECS. Comparing the two factor structures at two parenting points allowed us to produce more reliable results regarding the factor structure of the 19-item PSI-SF than would be possible with a single point. EFA revealed a three-factor structure, and the results of the model fit evaluation as well as validity and reliability tests indicated that a three-factor structure fit the 19-item PSI-SF better than the two-factor structure.

Factor 1 was characterized by the PD-SF subscale of the original 36-item PSI-SF, into which variables related to parenting role problems converged while Factor 2 reflected the DC-SF subscale, to which variables related to the child’s difficult behaviors contributed. Although the 19-item PSI-SF includes slightly different items from those used in the original 36-item PSI-SF and the other smaller-item versions, some of the items overlap with the findings of previous studies when it comes to the composition of Factors 1(PD-SF) and 2 (DC-SF). For example, the PD-SF of the 19-item PSI-SF consists of items 12 (Feeling incapable of handling things very well), 13 (Feeling unable to do what I like to do), 17 (Feeling alone without friends), and 19 (Unable to enjoy things as I used to)^3,10,11,16,19,22,23^. Likewise, the DC-SF consists of items 6 (Child cries or fusses rather often), 8 (Things that my child does bother me a lot), 9 (Child is easily upset by small things), and 10 (Child frequently puts demands on me)^10,11,16,19,22^. Excluding item 5 (Child rarely does things that make me feel good) from the DC-SF and item 7 (Child does not seem to smile much) from the PD-SF showed better scores for the model-fit indices of a three-factor structure. Previous studies that validated a three-factor structure of the PSI-SF suggested that items 5 and 7 significantly contribute to the PCDI-SF^10,11,14,16^, which the 19-item PSI-SF does not rely on. Factor 3 was explained by items 15 (My husband does not give me as much help as I expected) and 16 (More problems with my husband by having a child), which inquired about parenting stress associated with having a relationship with and help from the husband. This factor reflects the characteristic of the “Spouse” subscale in Abidin’s theory, which underpins the original PSI, although those variables contributing to the subscale were associated with father’s stress about children with disease^7,40^.

A unique feature of the 19-item PSI-SF is its reliance on the “Spouse” subscale instead of the PCDI-SF. Those two subscales are inherently different. An impaired parent–child relationship is not only a major parenting stress factor, but it can be associated with negative parenting, which may contribute to children developing problematic behavior^41^. Meanwhile, there is an association between support and cooperation from the husband and the mother’s health outcomes; for example, a mother who experiences a positive and stable relationship with her spouse can cope with stress better than those with a negative relationship^42^. Moreover, the involvement of the father in childcare is positively associated with the child’s social, emotional, and behavioral development^43,44^. In fact, a global trend in recent years indicates that fathers spend more time caring for their children than before^44^. In Japan, mothers have traditionally taken the chief role in parenting and childcare, while fathers have a relatively limited involvement compared with fathers in other countries, such as the USA, England, Sweden, Thailand, and South Korea^45,46^. Nevertheless, gradually over time, Japanese fathers are spending more time parenting, as a result of their increasing interest in participating in childcare and their spouse expecting the father’s involvement in childcare, particularly among the younger generations^45,46^. Given that the presence or absence of spousal support and a spousal relationship is associated with maternal parenting stress, the three-factor structure of the Japanese 19-item PSI-SF could be a valuable addition to the various PSI-SF versions already in use. However, using the 19-item PSI-SF for a parenting stress assessment in line with the “Spouse” subscale should be done with caution. For a more reliable assessment of maternal parenting stress, the 19-item scale should be re-established by selecting more relevant items for the “Spouse” subscale, such as “I do not spend a lot of time with my spouse due to my child’s disease” and “I no longer share with my spouse in doing many things^7^” and for the PD-SF, the DC-SF, or the “Spouse” subscales to account for the exclusion of items 5 and 7 for a better fit for the three-factor structure.

Maternal parenting stress level needs to be assessed from multiple aspects and using multiple criteria^20^. In this regard, incorporating alternative factors in existing versions of the PSI-SF instead of relying only on a two-factor structure will enable researchers and clinicians to conduct a more detailed and comprehensive assessment, thereby allowing them to ascertain potential factors contributing to maternal parenting stress. Using a multi-factor measure to obtain information in a rigorous manner may, in turn, help them to implement interventions to reduce or prevent maternal parenting stress.

The major strengths of this study are as follows. First, we utilized a large sample size (n = 79,282 and 75,831), which was ten times larger than that of previous studies. Second, the participants were recruited at 15 regional centers, including urban and rural areas across Japan, and therefore the results of this study can be regarded as representative of the Japanese population. Third, our findings were derived by comparing two different factor structure models and providing comparable model-fit index scores at two different parenting periods.

However, this study also has several limitations. First, our findings are for the 19-item PSI-SF and not its other forms. Support from a spouse plays various important roles in parenting, such as reducing poor parenting behaviors, decreasing parental distress, and helping cope with negative emotions and feelings^47^. However, our findings cannot be generalized to research and clinical trials using the full-scale PSI-SF to evaluate parenting stress due to the different variable construct of the PSI-SF. Therefore, further study is needed to evaluate the convergent validity of the 19-item PSI-SF used in the JECS by examining the correlation between it and the full-scale PSI-SF. Second, as discussed, the “Spouse” subscale of the 19-item PSI-SF was explained by two items only. To be used as an established parenting stress scale, the subscale should rely on at least three items, so that the factor stability becomes stronger as the number of variables (i.e., items) to be contained increases^48^. Finally, mothers, and not fathers, were the primary target of the 19-item PSI-SF employed in the JECS. Evaluating fathers’ parenting stress has proved challenging in several previous studies on parenting stress that used the PSI-SF^1,11,13,14,16^. Involving the male parent in such research can be difficult because the primary caregiver is often the mother in many different sociocultural contexts^12,14,21^. Nonetheless, further research involving fathers would help identify different causes of parenting stress with consideration of gender differences.

## Conclusion

This study supports a three-factor structure as most appropriate for a (19-item) PSI-SF for mothers from a general population^16^. Consistent results regarding the factor structure of the PSI-SF will be helpful to further studies and clinical trials of parenting stress in diverse populations as well as various social, economic, and cultural contexts.

## Data Availability

Data are unsuitable for public deposition due to ethical restrictions and the legal framework of Japan. It is prohibited by the Act on the Protection of Personal Information (Act No. 57 of 30 May 2003, amendment on 9 September 2015) to publicly deposit data containing personal information. Ethical Guidelines for Medical and Health Research Involving Human Subjects enforced by the Japan Ministry of Education, Culture, Sports, Science and Technology and the Ministry of Health, Labour and Welfare also restrict the open sharing of epidemiologic data. All inquiries about access to data should be sent to: jecs-en@nies.go.jp. The person responsible for handling enquiries sent to this e-mail address is Dr. Shoji F. Nakayama, JECS Programme Office, National Institute for Environmental Studies.
